# Seroprevalences of antibodies against ToRCH infectious pathogens in women of childbearing age residing in Brazil, Mexico, Germany, Poland, Turkey and China

**DOI:** 10.1017/S0950268820002629

**Published:** 2020-10-30

**Authors:** J. M. Warnecke, M. Pollmann, V. Borchardt-Lohölter, A. Moreira-Soto, S. Kaya, A. G. Sener, E. Gómez-Guzmán, L. Figueroa-Hernández, W. Li, F. Li, K. Buska, K. Zakaszewska, K. Ziolkowska, J. Janz, A. Ott, T. Scheper, W. Meyer

**Affiliations:** 1 Institute for Experimental Immunology, EUROIMMUN Medizinische Labordiagnostika AG, Lübeck, Germany; 2 Institute of Virology, Charité-Universitätsmedizin Berlin, Corporate Member of Freie Universität Berlin, Humboldt-Universität zu Berlin, and Berlin Institute of Health, Berlin, Germany; 3 Department of Medical Microbiology, İzmir Katip Çelebi University, Ataturk Training and Research Hospital, İzmir, Turkey; 4 Department of Science and Biotechnology, INOCHEM S.A. DE C.V., Col. San Miguel Ajusco, Mexico City, Mexico; 5 National Institute of Respiratory Diseases, Immunology and Autoimmunity Laboratory, Mexico City, Mexico; 6 EUROIMMUN Medical Diagnostics China Co., Ltd., Beijing, China; 7 EUROIMMUN Polska Sp. z.o.o., Wroclaw, Poland; 8 Diagnostic Laboratory CM Luxmed, Lublin, Poland; 9 Department of Laboratory Diagnostics, K. Marcinkowski Poznań University of Medical Sciences, Poznan, Poland; 10 Central Laboratory, Gynaecology and Obstetrics Clinical Hospital Poznan University of Medical Sciences, Poznan, Poland

**Keywords:** Antibodies, congenital infections, diagnostics, immune status, immunoblot, maternal infection, pregnancy, serology, seroprevalence, ToRCH

## Abstract

Determination of antibodies against ToRCH antigens at the beginning of pregnancy allows assessment of both the maternal immune status and the risks to an adverse pregnancy outcome. Age-standardised seroprevalences were determined in sera from 1009 women of childbearing age residing in Mexico, Brazil, Germany, Poland, Turkey or China using a multiparametric immunoblot containing antigen substrates for antibodies against *Toxoplasma gondii*, rubella virus, cytomegalovirus (CMV), herpes simplex viruses (HSV-1, HSV-2), *Bordetella pertussis*, *Chlamydia trachomatis*, parvovirus B19, *Treponema pallidum* and varicella zoster virus (VZV). Seroprevalences for antibodies against HSV-1 were >90% in samples from Brazil and Turkey, whereas the other four countries showed lower mean age-adjusted seroprevalences (range: 62.5–87.9%). Samples from Brazilian women showed elevated seroprevalences of antibodies against HSV-2 (40.1%), *C. trachomatis* (46.8%) and *B. pertussis* (56.6%) compared to the other five countries. Seroprevalences of anti-*T. gondii* antibodies (0.5%) and anti-parvovirus B19 antibodies (7.5%) were low in samples from Chinese women, compared to the other five countries. Samples from German women revealed a low age-standardised seroprevalence of anti-CMV antibodies (28.8%) compared to the other five countries. These global differences in immune status of women in childbearing age advocate country-specific prophylaxis strategies to avoid infection with ToRCH pathogens.

## Introduction

Among the main complications affecting the foetus such as congenital anomalies or birth defects, infections with pathogens of the ToRCH group are the most common causes. The term ToRCH encompasses infectious agents which can be transmitted to a child by vertical infection, either intrauterinally, sub partu or postnatally. ToRCH pathogens conventionally include *Toxoplasma gondii*, herpes simplex viruses types 1 and 2 (HSV-1 and HSV-2) and the teratogenic viruses cytomegalovirus (CMV) and rubella virus. Additional pregnancy-related pathogens are *Bordetella pertussis*, *Chlamydia trachomatis*, parvovirus-B19, *Treponema pallidum* and varicella zoster virus (VZV). Primary infections with some of the aforementioned ToRCH pathogens during pregnancy, especially during the first trimester, are associated with an increased risk of miscarriage, abortion, still birth, sterility, premature birth, congenital malformations, and foetal or neonatal transient or chronic disease. The risks of acquiring an infection during pregnancy and the consequences vary by pathogen [1]. In general, primary infections during pregnancy are substantially more damaging than secondary infections or reactivations. Compared with infections with one of the aforementioned ToRCH pathogens during pregnancy, ToRCH co-infections are associated with greater adverse impacts [2]. Details on transmission routes, symptoms and sequelae for each pathogen are reviewed for example in [3].

The likelihood of an infection with a ToRCH pathogen during pregnancy depends upon geographic region, preventive practices and on the mother's immune status against the specific virus. Depending on the respective infectious agent, having had a wildtype infection might confer immunity against reinfection (e.g. *T. gondii*, rubella virus, parvovirus B19), adaptive immunity which reduces after several years (e.g. *B. pertussis*), or limited immunity with the possibility of reinfections (e.g. HSV-1, HSV-2, CMV, *C. trachomatis*, *T. pallidum*). Successful vaccination might possibly confer immunity against infection with rubella virus and VZV.

Key aspects in the management of ToRCH infections are maternal prenatal screening, early recognition and treatment. For many of the ToRCH pathogens, prevention strategies or treatment are available. The detection of antibodies against ToRCH pathogens via serological testing before or at the beginning of pregnancy allows determination of the immune status of the mother. Serological testing is suitable to support the diagnosis of infection and distinguish between primary and secondary infections. For this purpose, maternal antibodies of class IgG are of specific interest as they can cross the placenta and provide passive immunity to the foetus. The results of antibody determination support the assessment of risks to a pregnancy and can guide the implementation of infection prophylaxis measures. Subsequently, close monitoring with further tests is part of prenatal care aiming at prevention of adverse foetal and obstetric outcomes. A routine full screening of the ToRCH panel is not recommended in low-risk asymptomatic pregnant women [4].

Generally, seroprevalences mirror exposure to pathogens which depend on environmental conditions such as tropical climate, season, vaccination coverage and dietary customs [5–8]. Specifically, seroprevalences of antibodies against ToRCH infectious agents are influenced by cultural aspects such as average maternal age, rate of kindergarten attendance and socioeconomic status. Previous research has been carried out to assess the seroprevalence of antibodies against single ToRCH infectious agents, for instance, in Venezuela, São Paulo State, Turkey, Istanbul, Croatia, northwest China, Japan and Ghana [6, 9–14]. Nevertheless, no study has compared differences in seroprevalences of the entirety of ToRCH pathogens across several continents.

The aim of the present study was to assess and compare seroprevalences of antibodies against ten ToRCH infectious agents (*T. gondii*, rubella virus, CMV, HSV-1, HSV-2, *B. pertussis*, *C. trachomatis*, parvovirus B19, *T. pallidum*, VZV) in serum samples from women of childbearing age residing in six countries across different climate zones. Data for this study were collected in Mexico, Brazil, Germany, Poland, Turkey and China. This comprehensive seroprevalence study will be useful to gynaecologists, midwives, epidemiologists, public health workers and laboratory personnel and will provide a better understanding of the overall distribution of ToRCH pathogens in the six countries.

A shortcoming of previous seroprevalence studies is the usage of different immunoassays, which might not be comparable because of the different antigens used in the assays, the conjugate and the assay format differing from one assay to another [15]. In this study, the maternal immune status was determined using a multiparametric immunoblot as a screening tool allowing simultaneous determination of specific antibodies of class IgG against ten different pregnancy-related pathogens. The particular advantage of the current approach is the usage of the same immunoassay in all six panels allowing unexcelled comparability of the results across countries.

## Methods

Diagnostic leftover samples from serum or plasma of healthy women of childbearing age independent of their pregnancy status (Table 1) were collected in Mexico City (Mexico), Salvador, São Paulo, Valença (Brazil), Lübeck (Germany), Wroclaw and Lublin (Poland), Izmir (Turkey), and Beijing, Sichuan, Tianjin, Shandong, Hebei and others (China). This study used archived samples and leftover samples after completion of all diagnostic measures. Samples were collected between 2006 and 2019, whereby the dates of collection differed between the countries (Table 1). Patient data were used anonymously. The samples from healthy Brazilian women originated from a case-control study [16]. The samples from healthy German women were collected via blood donation.

**Table 1.** Epidemiological characteristics of the included panels

Country	*N* samples	Age: mean ± standard deviation; range; 95% confidence interval	Sample collection
Mexico	100	30.6 ± 6.2; (19–41); (29.3–31.8)	July 2018–September 2018
Brazil	160	28.9 ± 6.6; (15–46); (23.4–29.9)	November 2015–May 2016
Germany	202	31.4 ± 8.0; (19–45); (30.6–32.9)	2006–2018
Poland	193	30.9 ± 4.7; (19–40); (30.2–31.6)	November 2018–February 2019
Turkey	97	26.6 ± 6.2; (20–41); (25.4–27.9)	October 2018
China	257	31.4 ± 4.7; (22–47); (30.8–32.0)^a^	October 2017–December 2017; August 2018–September 2018
Total	1009	30.3 ± 6.5; (15–47)^b^; (29.9–30.7)^b^	2006–2019

a Calculated for 232 participants (information was unavailable for 25 participants).

b Calculated based on 984 participants.

Samples from initially 1024 women were investigated for the presence of antibodies of the immunoglobulin class IgG against *T. gondii*, rubella virus, CMV, HSV-1, HSV-2, *B. pertussis*, *C. trachomatis*, parvovirus B19, *T. pallidum* and VZV using the EUROLINE Anti-TO.R.C.H. 10-Profile (IgG) (EUROIMMUN Medizinische Labordiagnostika AG, Germany). The test kit contains line blots coated with parallel lines of highly purified antigens (Table 2). Results were included if control bands indicated a correctly performed test leading to 1009 samples that were considered in the analysis. The test was performed according to the instructions of the manufacturer. The strips were evaluated automatically using the EUROLineScan software (EUROIMMUN). No or very weak band signal intensities were interpreted as negative results by the software and double-checked by a lab technician, while medium to strong band signal intensities were interpreted as positive bands. Borderline results showing weak band signal intensities were considered as negative. Seroprevalences were averaged per ToRCH pathogen and visualised including standard deviations to reveal the extent of deviation across countries. Country-specific seroprevalences were standardised for age based on the WHO's world standard population, whose age groups were restricted to those present in the current dataset. For this, 984 samples with available age information were used (Table 1). For every country, prevalences were calculated for seven age groups (15–19, 20–24, 25–29, 30–34, 35–39, 40–44 and 45–49 years) and weighted according to the scaled standard population. Weighted means across age groups with 95% confidence intervals were calculated. Seroprevalences of antibodies against ten ToRCH pathogens were compared between countries. The data originating from Brazil have been included in a previous publication by Moreira-Soto *et al*. (2018) [17].

**Table 2.** Antigens included in the EUROLINE Anti-TO.R.C.H. 10-Profile (IgG)

Pathogen	Antigen
*Toxoplasma gondii*	Inactivated lysates of *T. gondii* tachyzoites
Rubella virus	Inactivated lysates of Vero cells infected with the HPV-77 strain of rubella virus
Cytomegalovirus	Recombinant phosphoproteins of cytomegalovirus, expressed in *E. coli*
Herpes simplex virus type 1	Affinity-purified native glycoprotein C1 (gC1)
Herpes simplex virus type 2	Affinity-purified native glycoprotein G2 (gG2)
*Bordetella pertussis*	Antigen preparation of pertussis toxin (PT) from *B. pertussis*
*Chlamydia trachomatis*	Recombinant major outer membrane protein (MOMP) of *C. trachomatis*
Parvovirus B19	Recombinant viral protein 1 (VP1) of human parvovirus B19
*Treponema pallidum*	Recombinant lipoproteins of *T. pallidum*, expressed in *E. coli*
Varicella zoster virus	Native glycoproteins of varicella zoster virus

## Results

**Fig. 1.**
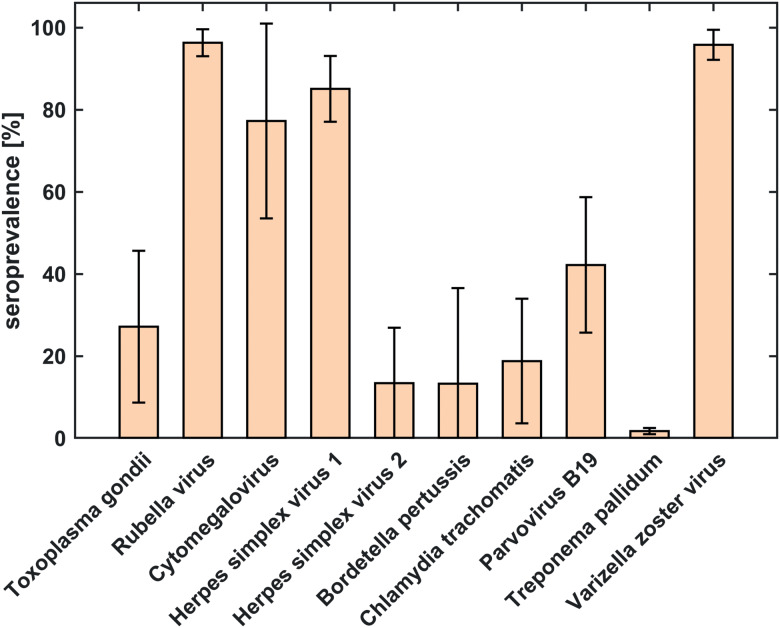
Comparison of seroprevalences of antibodies against 10 ToRCH pathogens averaged over six countries based on serum samples from 1009 women of childbearing age. The error bars represent standard deviation and indicate the amount of deviation across counties.

Seroprevalences averaged across countries (Fig. 1, Table 3) were highest for antibodies against rubella virus (96.3%), VZV (95.9%), HSV-1 (85.1%) and CMV (77.3%). The lowest overall seroprevalence was observed for *T. pallidum* (1.7%). The largest differences across countries were observed for antibodies against CMV (±23.7% standard deviation (s.d.)) and *B. pertussis* (±23.3% s.d.). Deviations across countries were smallest for antibodies against *T. pallidum* (±0.7% s.d.) and rubella virus (±3.3% s.d.).

**Table 3.** Age-standardised country-specific seroprevalences of antibodies against 10 ToRCH pathogens were calculated based on samples with available age information (n = 984) and reported as mean percentage and 95% confidence interval. Mean and standard deviation (s.d.) across countries for each pathogen were calculated based on all available samples (n = 1009).

Pathogen	Mexico	Brazil	Germany	Poland	Turkey	China	Mean	Standard deviation
Seroprevalence of IgG antibodies against								
*T. gondii*	31.4 (6.4–56.3)	56.2 (45.6–66.9)	39.5 (13.5–65.6)	22.3 (7.2–37.4)	34.7 (10.3–59.2)	0.5 (0.0–1.4)	27.2	18.5
Rubella virus	99.0 (96.6–100.0)	97.4 (94.3–100.0)	97.9 (95.2–100.0)	99.8 (99.2–100.0)	94.1 (86.1–100.0)	83.2 (65.7–100.0)	96.3	3.3
CMV	79.9 (68.2–91.5)	98.1 (94.6–100.0)	28.3 (10.3–46.3)	68.8 (51.5–86.0)	98.4 (93.9–100.0)	91.9 (84.3–99.6)	77.3	23.7
HSV1	75.6 (60.3–90.8)	95.7 (92.5–98.8)	62.5 (36.5–88.4)	85.7 (70.3–100.0)	94.7 (87.7–100.0)	87.9 (79.1–96.7)	85.1	8.0
HSV2	10.7 (0.0–21.5)	40.1 (27.5–52.7)	8.3 (0.0–17.3)	4.1 (0.0–9.3)	2.1 (0.0–6.5)	7.2 (0.7–13.6)	13.4	13.5
*B. pertussis*	0.7 (0.0–2.5)	56.6 (47.3–66.0)	22.2 (0.0–46.9)	1.2 (0.0–3.2)	1.6 (0.0–6.1)	0.2 (0.0–0.8)	13.3	23.3
*C. trachomatis*	14.8 (6.1–23.5)	46.8 (40.5–53.0)	10.9 (3.8–18.0)	7.4 (0.0–15.9)	13.9 (0.0–31.6)	18.8 (4.5–33.1)	18.8	15.2
Parvovirus B19	50.1 (37.6–62.5)	31.1 (17.6–44.7)	46.7 (28.1–65.4)	65.1 (40.6–89.6)	50.6 (36.2–64.9)	7.7 (1.1–14.4)	42.2	16.5
*T. pallidum*	1.4 (0.0–3.7)	0.9 (0.0–2.2)	1.1 (0.0–2.4)	1.7 (0.0–5.0)	2.3 (0.0–7.1)	1.0 (0.0–2.2)	1.7	0.7
VZV	98.7 (95.4–100.0)	94.5 (88.6–100.0)	99.4 (98.1–100.0)	98.1 (95.7–100.0)	98.6 (96.2–100.0)	92.3 (84.5–100.0)	95.9	3.7

**Fig. 2.**
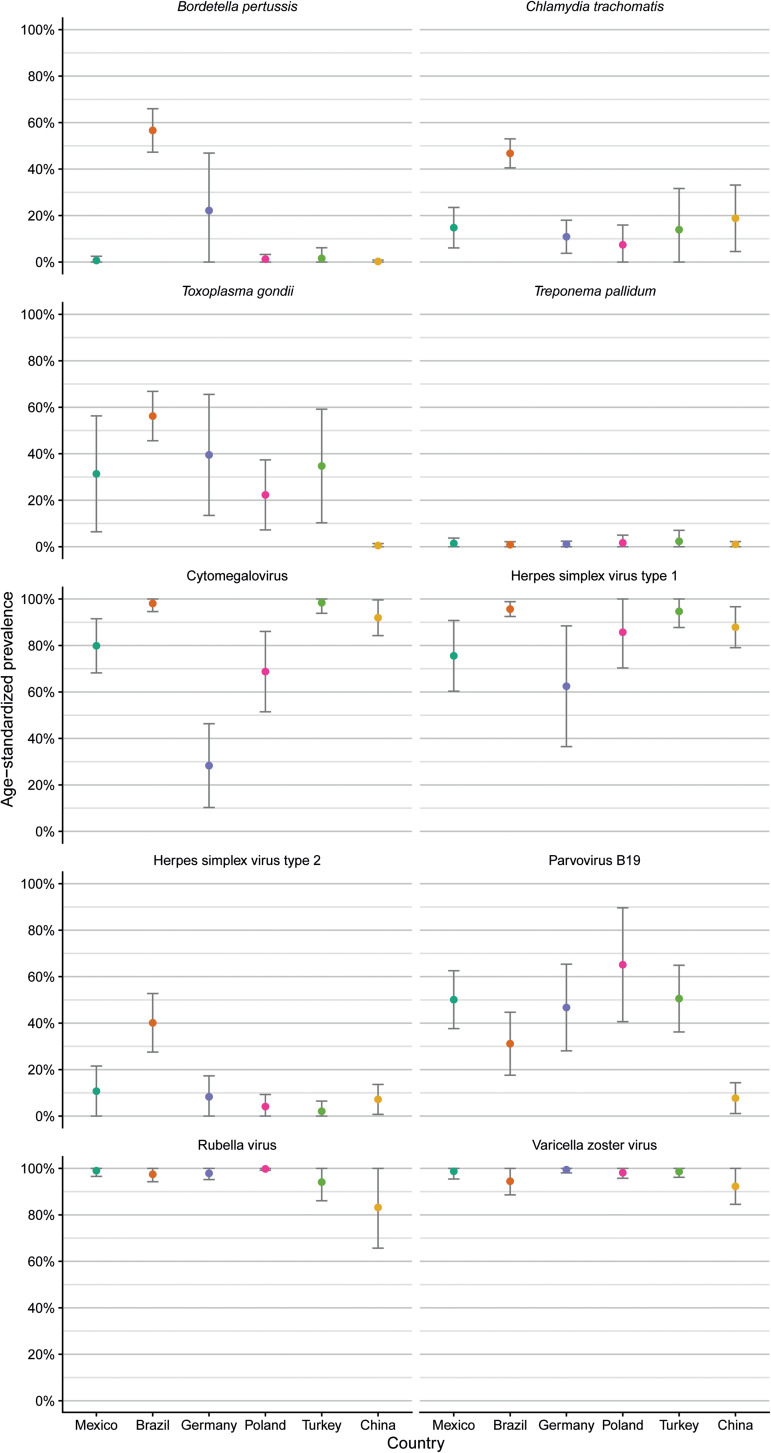
Comparison of age-standardised seroprevalences of antibodies against 10 ToRCH pathogens in six countries based on serum samples with available age information (n = 984) and reported as mean percentage and 95% confidence interval.

Age-standardised seroprevalences for antibodies against *T. pallidum* showed consistently low levels across countries (Fig. 2, Table 3). Age-standardised seroprevalences for antibodies against VZV and rubella virus were consistently high across countries, although seroprevalences for antibodies against rubella virus showed large 95% confidence intervals for samples from Turkey and China compared to the other four countries indicating large differences between age groups.

Mean age-adjusted seroprevalences for antibodies against HSV-1 were >90% with small confidence intervals in samples from Brazil and Turkey, whereas the other four countries showed lower mean age-adjusted seroprevalences (range 62.5–87.9%) with large 95% confidence intervals indicating pronounced differences across the seven age groups (Table 3).

Samples from Brazilian women showed elevated age-standardised seroprevalences of antibodies against HSV-2 (40.1%), *C. trachomatis* (46.8%) and *B. pertussis* (56.6%) compared to the other five countries. Age-standardised seroprevalences of anti-*T. gondii* antibodies (0.5%) and anti-parvovirus B19 antibodies (7.5%) were low in samples from Chinese women, compared to the other five countries. Samples from German women revealed a low age-standardised seroprevalence of anti-CMV antibodies (28.8%) compared to the other five countries.

## Discussion

### Summary

The primary objective of the current study was to present an overview about seroprevalences of ten ToRCH pathogens (*T. gondii*, rubella virus, CMV, HSV-1, HSV-2, *B. pertussis*, *C. trachomatis*, parvovirus B19, *T. pallidum*, VZV) based on 1009 serum samples from women of childbearing age residing in six countries (Mexico, Brazil, Germany, Poland, Turkey and China). The most obvious findings to emerge from this comparative analysis were elevated seroprevalences of antibodies against *T. gondii*, HSV-2, *B. pertussis* and *C. trachomatis* in samples from Brazilian women. Across the six countries, Chinese women had the lowest seroprevalence of antibodies against *T. gondii* and parvovirus B19, whereas German women had the lowest seroprevalence of anti-CMV antibodies. As expected, the observed seroprevalences speak in favour of global differences in immune status advocating country-specific infection prophylaxis strategies.

To the best of our knowledge, the current study is the first to provide seroprevalences of antibodies against multiple pathogens of the ToRCH complex in women of childbearing age residing in Mexico, Germany and Poland. The discussion of the observations presented in this study is largely based on comparison to previous research studying one of the ToRCH pathogens and is therefore ordered by pathogen.

#### Toxoplasma gondii

Of the Brazilian women evaluated for the presence of anti-*T. gondii* antibodies, 59% showed IgG-positive results, which indicates a past infection/chronic phase. The remaining women were seronegative for IgG, representing a population at risk of developing toxoplasmosis throughout the gestational period. These results were very similar to data found in other regions of Brazil [18, 19].

These results indicate that Brazil has a relatively high seroprevalence of antibodies against *T. gondii* in comparison with the other countries. The high anti-*T. gondii* IgG seroprevalence found there may be explained by climatic, geographic and socioeconomic characteristics of these countries.

Seroprevalences of antibodies against *T. gondii* in China were reported to range between 5% based on a survey conducted between 1988 and 1992 and 25% in provinces with particular eating habits of the respective resident ethnic group [20]. Between 2001 and 2004, the mean seroprevalence of 15 Chinese provinces was 8% [21]. The present finding of the seroprevalence of antibodies against *T. gondii* in Chinese women of 1% seems to be rather low when compared with previous studies. This might be explained by the recentness of the study. In Turkish women, the observed seroprevalence for anti-*T. gondii* IgG (26%) was not in agreement with an earlier study reporting 48% among pregnant women [22]. Explanations for this disagreement might be that the study by Tamer *et al*. and the present one were performed roughly 10 years apart from each other, assessed women from two far-off regions of Turkey (Kocaeli *vs.* Izmir) and used different techniques (ELISA *vs.* immunoblot).

#### Rubella

Various inoculation strategies have been employed worldwide to prevent rubella infections. Compared with evidence from 2010, the seroprevalence of anti-rubella antibodies increased from 93% to 98% in Brazilian women [19]. In several countries, e.g. Latin America and Europe, vaccination campaigns against rubella have contributed to these high immunisation rates. The percentages of women at risk for congenital transmission of rubella were very low in all tested countries.

In Turkish women, the observed seroprevalence for anti-rubella IgG (94%) were in accordance with previous studies reporting 94–96% [22–24]. Since rubella vaccine was not incorporated into the national immunisation programme in Turkey until 2006, seropositivity was probably caused by natural infections.

#### CMV

The results imply that about 62% of the German women are susceptible to infection with CMV. A recent study investigated changes in CMV IgG seroprevalence of the patients of a German urban hospital over a time period of 30 years and observed a continuous seroprevalence around 65% in women of childbearing age [25]. The distribution between different age groups among the women revealed the highest seroprevalence in the youngest age group (16–20 years of age), that is declining up to the age group 31–35 years of age, followed by a continuous increase with progressing age [25]. Possible reasons for the lower CMV seroprevalence in Germany in this study could be the tendencies towards smaller households with fewer young children as possible sources of infection during the last decades [25]. In Turkish women, the observed seroprevalence for anti-CMV (99%) were in accordance with previous studies reporting 94–97% [22–24]. The higher anti-CMV seroprevalences observed in Mexico, Brazil, Turkey and China might be related to social factors like earlier and more extensive involvement of women of childbearing age in the care of potentially infectious toddlers and infants.

#### HSV-1

Seroprevalences for anti-HSV-1 antibodies were around 90% in the present study. A seroprevalence study from 2010 found that anti-HSV-1 seroprevalence was 72% but varied with age and among Brazilian regions [26]. The observation of age dependency is contrary to a recently published study in a Chinese panel suggesting that the seroprevalence of anti-HSV-1 antibodies is not affected by age [9]. In Brazilian women who participated in the cervical cancer screening program, the prevalence of anti-HSV-1 was 23% [27]. In a study from 1996, the prevalence of anti-HSV-1 antibodies in the general population was 67.2% and significantly associated with age and geographic region, but not with gender, socio-economic condition, prior history of STDs or anti-HSV-2 antibodies [26]. Reasons for a high prevalence of HSV-1 positivity might possibly be related to sexual practices in regard to oral-genital contact or receptive oral sex [28].

#### HSV-2

The results of seroprevalences of antibodies against HSV-2 being 39% in serum samples from Brazilian women were higher than those obtained in 1994 being 29.1% among a group at low risk for acquiring sexually transmittable diseases [29]. A cross-sectional study including subjects aged 1–40 years from the general population in four different geographical areas in Brazil between 1996 and 1997 reported age-adjusted seroprevalences of anti-HSV-2 antibodies in Brazil to be 11%, to increase with years of age and a higher increase was observed among adolescents and young adults [26]. Age, geographic region, prior history of sexually transmissible diseases and seropositivity for HSV-1 were significant and independent factors for HSV-2 infection [26]. In 2000, a study detected a prevalence of 42% for anti-HSV-2 antibodies in 181 middle-aged Brazilian women participating as control subjects in two cervical cancer studies [30]. Risk factors for HSV-2 positivity included a history of casual partners, no reported condom use, a history of smoking for more than 20 years, living in an urban or semi-urban residence was and a report of a partner who occasionally or frequently had other sexual partners [30]. HSV-2 prevalence is highest in Africa and the Americas, varies overall and by age markedly by country, region within country and population subgroup [31]. Age-specific HSV-2 prevalence is usually higher in women than men and in populations with higher risk sexual behaviour [31].

#### Bordetella pertussis

The highly contagious *B. pertussis* is distributed worldwide. The disease occurs sporadically or epidemically, but season and climate have no influence on its frequency. Vaccination is available and recommended. Compared with the other five countries, the seroprevalence of antibodies against *B. pertussis* was found to be highly elevated (59%) in Brazilian women and especially high in women from 26 to 30 years of age. These results may be related to a pertussis re-emergence in Brazil that reached epidemic levels from 2012 to 2014 [8].

#### Chlamydia trachomatis

Comparison of our findings with those of two other studies confirms the seroprevalence of antibodies against *C. trachomatis* in Turkish women to be around 10–12% [32, 33]. However, an unexpected and unprecedented high seroprevalence of 52% for antibodies against *C. trachomatis* has been detected using ELISA in 1115 women residing in a rural area of Ankara [34], which was interpreted by the authors as a sign of an increase in the prevalences of sexually transmitted diseases in the Turkish population.

#### Parvovirus B19

The results regarding seroprevalence for antibodies against parvovirus B19 from Mexico, Brazil, Germany, Poland and Turkey ranging from 36 to 55% are broadly consistent with earlier reports from India, Tanzania, Iran and Japan [35–38]. A study by Mossong *et al*. determined age-specific anti-B19 seroprevalence profiles in five European countries (Belgium, England and Wales, Finland, Italy, Poland) by testing blood samples from large serum banks for the presence of IgG antibodies against parvovirus B19 using the same assay for all samples [39]. Age-specific seroprevalence profiles followed broadly similar patterns across the five countries, showing a rapid increase of seroprevalence in childhood, followed by a plateau in young adults and then another increase in age groups about >25–30 years. Upon assessing the total burden of parvovirus B19 among pregnant women, the highest risks of acquiring an infection during pregnancy were estimated for Poland and Finland [39]. In contrast, a recently published study suggested that the seroprevalence of ToRCH pathogens is not affected by age [9]. In the present study, the seroprevalence of antibodies against parvovirus B19 in Poland were among the highest, reflecting low risk of acquiring infection when compared to, e.g. China and Brazil. The low seroprevalence of 12% in Chinese women in the present study was unanticipated.

#### Treponema pallidum

Seroprevalences of antibodies against *T. pallidum* from our study (1–3%, 1% in Brazil) were consistent with previous studies showing low (0–3%) seroprevalence in Brazilian adult women [19, 40]. The worldwide low seroprevalence rates suggest that many expecting women are at risk to contract syphilis and should be advised how to apply control measures to prevent congenital syphilis.

#### VZV

Seroprevalences of antibodies against VZV were on comparably high levels across countries (range: 90–99%). Vaccination campaigns against VZV in e.g. Latin America and Europe have contributed to the high immunisation rates. Hence, the percentages of expecting mothers at risk for congenital transmission of VZV were very low in the tested countries. In German women, seropositivity for VZV was probably elicited by natural infection since vaccinations were only recommended for children from 2004 onwards.

As mentioned before, the interpretation of and the agreement between published studies assessing maternal immunity is complicated by the use of different serological assays. The multiparametric lineblot EUROLINE Anti-TO.R.C.H. 10-Profile (IgG) could be a solution providing an extended assessment of several ToRCH pathogens and rectifying the incomparability between seroprevalence studies. A future goal thus could be to collect data on seroprevalence in each country, using the same, accepted test system.

Relevant for diagnostic laboratories is the acknowledgement of the efficacy of the multiparametric immunoblot as an ideal tool for a comprehensive assessment of maternal immunity. The EUROLINE Anti-TO.R.C.H. 10 Profile (IgG) offers the most extensive profile for the detection of IgG antibodies against ten pregnancy-relevant infectious agents on one test strip and in a single incubation. Compared with using a test system comprising only the five standard ToRCH pathogens, utilisation of this multiparametric immunoblot, including five additional, non-standard ToRCH pathogens, yields twice the information, while time and effort to conduct the test are comparable. Furthermore, due to the option of fully automatable processing of immunoblot tests with the EUROBlotOne (EUROIMMUN) combined with automated evaluation using the EUROLineScan software (EUROIMMUN), the test is suitable for efficient analysis of a large number of samples as is required for comprehensive seroprevalence studies. Automated testing minimises individual interpretation errors, which leads to a high level of standardisation.

### Limitations

Evidence of the present seroprevalence study is limited by the number of included serum samples and countries of sample origin. Nevertheless, no other seroprevalence study has ever compared immune statuses of women residing in six countries. The participants were included independent of their pregnancy status, i.e. at the time of blood drawing the women were nulliparous, nulligravidae, (primi)gravidae or multigravidae. Pregnant participants received medical attendance independent of the current study and results of the serological assessment were not intended for individual diagnoses. Direct comparisons of results of the present study with other studies should be made with caution due to differences in the populations studied, age composition and laboratory methodology. This assessment of seroprevalence is an evaluation of the status quo, whereas alterations in prevalence over time need to be monitored using repeated assessments. Here, seroprevalences were determined based on specific IgG antibodies. Since testing of acute infections based on antibodies of class IgM was not performed, primary infection acquired during the weeks preceding the testing could not be ruled out. Furthermore, immune responses elicited by vaccination and those elicited by natural infection with wild-type pathogens could not be differentiated by the used immunoassay. Vaccination plans are country-specific and influence the presence of antibodies against VZV, *B. pertussis* and rubella virus. In this study, information about the women's vaccination status or medical history was not available. Unfortunately, information on age of the Chinese women was not available for all participants. General limitations of serodiagnostic assays that also limit the present results include interpretation of borderline outcomes and inter-assay variability.

### Outlook

The seroprevalences of ToRCH pathogens presented in this study can serve as a reference providing an informative basis for individual infection prophylaxis measures to patients. Determination of IgG antibodies against ToRCH pathogens can be performed for serological confirmation of the immune status of expectant mothers and assessing the risks to the pregnancy.

Based on the evidence presented here, the currently implemented national public health policies could be re-evaluated after conduction of larger studies designed to generate more representative local data. Alternatively, the data presented here could serve as a basis for the development of a vaccination strategy against ToRCH infectious agents. In an effort to do so, different needs and access to medical care for different geographic and resource settings need to be acknowledged [41]. In the same vein, awareness of and knowledge about infectious agents could be improved by educating women of childbearing age on how to prevent mother-to-child infections [14].

Further research should be undertaken to evaluate local differences in seroprevalences within a country as well as differences in seroprevalences between age groups of the same country, which requires the collection of a large number of serum samples.

In future, the ToRCH complex might be extended by further pathogens. A potential candidate could be Zika virus (ZIKV), due to the similarities of its sequelae to the complications associated with the ToRCH pathogens [41, 42]. Maternal ZIKV infection during pregnancy is usually asymptomatic for the mothers, but can induce congenital malformations in the foetus [43], i.e. congenital Zika syndrome including skull and brain deformities, ocular abnormalities, arthrogryposis and spasticity [44]. Since the outbreak of ZIKV infections in Latin America in 2015–2016, the local incidence of neonatal microencephaly has dramatically increased [17]. In the circumstances of an outbreak of ZIKV infections, TORCH pathogens might be disregarded as causes of congenital malformations or pre- and postnatal complications [17]. Therefore, combined testing of ZIKV and pathogens of the ToRCH complex should be carried out to determine the immune status of women residing in regions with emerging arboviruses.

## Data Availability

The data that support the findings of this study are available from the authors [VBL or JMW] upon reasonable request.
